# Microbiota and mycobiota in chronic inflammatory demyelinating polyneuropathy: clinical and pathophysiological correlations

**DOI:** 10.3389/fnmol.2026.1802462

**Published:** 2026-06-03

**Authors:** Szymon Andrusiów, Anita Brzoza, Piotr Łacina, Marta Dratwa-Kuzmin, Justyna Raczkowska, Dorota Kujawa, Katarzyna Romańczuk, Marta Gancarek, Patrycja Bochen, Edyta Dziadkowiak, Bogumiła Szponar, Łukasz Łaczmański, Magdalena Koszewicz, Katarzyna Bogunia-Kubik

**Affiliations:** 1 Department of Neurology, Faculty of Medicine, University Centre of Neurology and Neurosurgery, Wrocław Medical University, Wrocław, Poland; 2 Laboratory of Genomics and Bioinformatics, Hirszfeld Institute of Immunology and Experimental Therapy, Polish Academy of Sciences, Wrocław, Poland; 3 Laboratory of Clinical Immunogenetics and Pharmacogenetics, Hirszfeld Institute of Immunology and Experimental Therapy, Polish Academy of Sciences, Wrocław, Poland; 4 Laboratory of Medical Microbiology, Hirszfeld Institute of Immunology and Experimental Therapy, Polish Academy of Sciences, Wrocław, Poland; 5 Clinical Neurophysiology Laboratory, Faculty of Medicine, University Centre of Neurology and Neurosurgery, Wrocław Medical University, Wrocław, Poland

**Keywords:** chronic inflammatory demyelinating polyneuropathy (CIDP), gut microbiota, gut mycobiota, IL18 polymorphisms, short chain fatty acids (SCFAs)

## Abstract

**Introduction:**

Chronic inflammatory demyelinating polyneuropathy (CIDP) is an immune mediated neuropathy with incompletely defined triggers and no validated biomarkers. We investigated associations between bacterial and fungal communities, short chain fatty acids (SCFAs), and *IL18* promoter variation with clinically/electrophysiologically relevant measures.

**Methods:**

We enrolled 32 treatment naive CIDP patients and 15 healthy controls. Stool, serum and cerebrospinal fluid (CSF) samples were collected. Disability was assessed using the clinical scale and related to nerve conduction study (NCS) parameters. *IL18* promoter variants (rs187238, rs1946518, and rs1946519) were genotyped in haplotype based comparisons. SCFAs were quantified in stool, serum, and CSF. Faecal and CSF microbiota and mycobiota were profiled by amplicon sequencing of the 16S rRNA V3 V4 region and the ITS1 region, respectively. Bioinformatic processing and diversity analyses were performed in QIIME2. Alpha diversity and beta diversity metrics were computed across taxonomic levels. Differential abundance testing was conducted with ANCOM BC2.

**Results:**

CIDP was characterised by increased faecal bacterial alpha diversity and significant shifts in beta diversity relative to controls. Diversity patterns differed across clinical subgroups, including diabetes status and disability severity. Higher faecal bacterial diversity metrics correlated with greater NCS abnormalities, whereas associations with beta diversity measures were weaker. Taxonomic analyses identified enrichment of *Hominisplanchenecus_A faecis* and depletion of *Lawsonibacter sp000177015* in CIDP. SCFAs concentrations correlated with bacterial diversity indices, and *IL18* haplotypes were associated with distinct microbial signatures. Faecal mycobiota showed no robust case control differences in overall diversity. CSF sequencing revealed a low biomass bacterial signal and diversity metrics correlated with neurophysiological measures.

**Discussion:**

We confirmed the increased gut microbiome alpha diversity in CIDP patients, as observed in previous research. Our findings suggest significant alterations in both gut microbiota and mycobiota during the course of CIDP, which correlate with clinical, electrophysiological, and laboratory parameters.

## 1 Introduction

In recent years, there has been growing interest in the potential influence of the gut microbiome on neurological disorders (Cryan et al., 2020). The role of the gut microbiota in autoimmune disease pathogenesis remains debated. However, it appears to influence autoimmune responses throughout the body and to drive inflammatory reactions in the gut wall (Qiu et al., 2022; Romero et al., 2022; Zeng et al., 2022). Intestinal dysbiosis is also recognized as a contributing factor in the pathophysiology of neurodegenerative diseases (Cheng et al., 2023; Solch et al., 2022). For example, faecal microbiota transplantation (FMT) has shown promising effects in certain neurological conditions, such as Parkinson’s disease and cerebrovascular disease (Koszewicz et al., 2021a). In diabetic neuropathy, FMT was associated with an improved clinical phenotype alongside an increase in butyrate-producing gut bacteria (Vendrik et al., 2020; Yang J. et al., 2023). Moreover, dietary interventions such as intermittent fasting may affect gut microbiota composition and, in turn, affect for systemic immunity and autoimmunity in the central nervous system (CNS) (Cignarella et al., 2018). Defining what constitutes unequivocal dysbiosis versus variation within the healthy range remains challenging. The NIH Human Microbiome Project, including its second, integrative phase, has provided extensive resources and reference frameworks (Proctor et al., 2019). Current large-scale cohorts are extending this work to refine healthy baselines and disease-linked deviations (e.g., Le French Gut, the Microsetta Initiative/American Gut, the Dutch Microbiome, UK Biobank microbiome sub-studies). Within this landscape, a commonly used descriptive framework remains the three gut enterotypes, dominated by *Bacteroides*, *Prevotella*, or *Ruminococcus* (Arumugam et al., 2011). Individual studies indicate the contribution of specific types of bacteria to the autoimmune response resulting in myasthenia gravis and Guillain-Barré syndrome (Cao et al., 2024; Zhang et al., 2024). Other studies show dysbiosis in chronic inflammatory demyelinating polyneuropathy (CIDP) and disturbances in the gastrointestinal bacterial metabolome (Fu et al., 2023). However, studies assessing the role of dysbiosis in the pathogenesis and course of CIDP are currently lacking. Evidence on the gut mycobiome in autoimmunity is limited. In neurology, small studies in multiple sclerosis (MS) suggest altered fungal diversity and taxonomic shifts with links to inflammatory markers, but findings are not yet consistent (Otaegui-Chivite et al., 2025). In CIDP, very limited profiling of mycobiome has not shown clear abnormalities in diversity or community structure. These null results should be viewed cautiously given small cohorts, limited taxonomic resolution, and confounding by diet, antibiotics, and antifungals (Verhasselt et al., 2023). Intestinal dysbiosis (bacterial or fungal) may be a risk factor for autoimmune diseases through multiple mechanisms. The main lines of research consider modulations of gut-associated lymphoid tissues (GALT), changes in intestinal barrier permeability and the associated increase in bacterial metabolites entering the bloodstream (Levy et al., 2015; Qiu et al., 2022; Rea et al., 2016; Ruff et al., 2020).

Short-chain fatty acids (SCFAs) are metabolites produced by gut bacteria that can affect human physiology via multiple mechanisms. SCFAs serve as an important energy source for intestinal epithelial cells, particularly colonocytes, help stabilize the intestinal barrier by supporting epithelial integrity and modulating mucosal immune responses, and exert immunomodulatory effects on intestinal immune cells (Blacher et al., 2017). SCFAs have also been implicated in neurodegenerative conditions; for example, they may contribute to the development of Parkinson’s disease and Alzheimer’s disease by modulating microglial activity (Colombo et al., 2021). By contrast, patients with MS, an inflammatory disease of the CNS, have been found to exhibit reduced SCFA levels (Montgomery et al., 2024). Evidence for SCFA involvement in inflammatory neuropathies remains scarce, and their ability to enter the cerebrospinal fluid (CSF) has so far only been shown in animal models; to date, limited measurements in human CSF have been reported (Deng et al., 2019). One immune mediator of particular interest is interleukin-18 (IL-18), a cytokine activated via the NOD-like receptor pyrin domain containing 6 (NLRP6) inflammasome. IL-18 can substantially influence gut homeostasis by affecting intestinal barrier integrity, mucosal inflammation, and the composition of the microbiome (Seregin et al., 2016; Yasuda et al., 2019). This molecule also exerts broad pleiotropic effects: it functions as a pro-inflammatory cytokine and influences neuronal survival by upregulating brain-derived neurotrophic factor (BDNF) expression (Ihim et al., 2022; Yasuda et al., 2019; Zhou et al., 2014). Despite extensive research, the exact role of IL-18 in autoimmune disorders remains unclear, with many findings proving contradictory (Elinav et al., 2011; Nowarski et al., 2015; Salcedo et al., 2010). Nevertheless, several studies have reported elevated IL-18 levels in a range of autoimmune conditions, including those affecting the CNS (Ihim et al., 2022). There are also isolated observations suggesting that IL-18 may play a role in the pathogenesis of inflammatory demyelinating neuropathies (Jander and Stoll, 2001). In light of these findings, two functional polymorphisms in the *IL18* promoter region (-137G/C, (rs187238) and -607C/A, (rs1946518), have gained attention. Notably, rs1946518 is in linkage disequilibrium (LD) with rs1946519, which is therefore often considered in haplotype-based analyses. These genetic variants can modulate the transcription of IL18, thereby shifting the cells’ cytokine secretion capacity and the overall inflammatory baseline of the individual. Notably, the impact of these *IL18* promoter variants can depend on the population context; yet numerous studies have repeatedly linked certain *IL18* genotypes or haplotypes to altered IL-18 levels and specific clinical features in autoimmune disease. Accordingly, the rs187238 and rs1946518 variants are increasingly regarded as potential determinants of CIDP severity and clinical presentation (An et al., 2008; Dziedziejko et al., 2012; Ramezanzadeh et al., 2024).

CIDP is an uncommon, immune-mediated neuropathy, with an estimated prevalence on the order of only 1.6–8.9 per 100,000 people in various populations (Iijima et al., 2008; Laughlin et al., 2009; Mygland and Monstad, 2001). The direct effector role in peripheral myelin injury is thought to be mediated largely by classically activated (M1) macrophages (Msheik et al., 2022). Cytotoxic CD8^+^ T cells, CD4^+^ helper T cells (Th1 and Th17), and the complement system are also implicated (Koike et al., 2018; Schneider-Hohendorf et al., 2012; Vallat et al., 2020; Yang et al., 2015). Numerous other mediators participate in the inflammatory cascade, including both pro- and anti-inflammatory cytokines and, likely, autoantibodies (Marí-Alexandre et al., 2023; Moritz et al., 2021; Wolbert et al., 2020). The pathogenic core appears to be a self-reinforcing feed-forward loop. Macrophages, once myelin is opsonised via complement (especially around the nodes of Ranvier), directly phagocytose the myelin sheath. These macrophages can express major histocompatibility complex (MHC) class II and co-stimulatory B7 molecules, enabling them to present myelin antigens to Th1/Th17 cells and possibly B cells, which in turn further activate macrophages, amplifying the cycle (Bour-Jordan et al., 2013; Chi et al., 2010; Msheik et al., 2022; Wolbert et al., 2020; Xue, 2025; Yang et al., 2015). The chronicity of inflammation is thought to be sustained in part by tissue-resident macrophages that acquire a pro-inflammatory M1-like program (Msheik et al., 2022). A preceding breakdown of both central and peripheral tolerance is presumed necessary, yet the initiating trigger of autoimmunity remains unclear (Su et al., 2012). To date, no specific biomarker has been validated that reliably predicts prognosis, treatment response, or assists diagnosis. Moreover, antibody-mediated conditions previously linked to CIDP have been reclassified as distinct disease entities, so-called nodopathies and anti-myelin-associated glycoprotein (MAG) neuropathy (Broers et al., 2023; Ogata, 2020; Pascual-Goñi et al., 2024; Querol et al., 2017; Van den Bergh et al., 2021). One promising line of inquiry is the evaluation of inflammatory cytokines as potential biomarkers and pathogenic factors. In particular, IL-2 and TNF-α have garnered interest due to their broad immunomodulatory actions and suggested involvement in CIDP pathogenesis (Dziadkowiak et al., 2021; Misawa et al., 2001). Clinically, the disorder most often presents with symmetric weakness involving both proximal and distal muscle groups accompanied by sensory symptoms, and the overall course is progressive (Van den Bergh et al., 2021). However, several atypical CIDP variants have been described that differ in their pattern of nerve involvement or disease course (Doneddu et al., 2019; Menon et al., 2021). According to the 2021 EAN/PNS criteria, the main CIDP variants include distal CIDP, formerly described as distal acquired demyelinating symmetric neuropathy (DADS), as well as motor, sensory, focal, and multifocal CIDP. Diagnosis relies on the clinical examination together with nerve conduction studies (NCS) to demonstrate a demyelinating pattern in peripheral nerves (Van den Bergh et al., 2021). The prevailing emphasis is on the role of patient assessment using appropriate clinical scales, such as the Inflammatory Neuropathy Cause and Treatment (INCAT) scale or Inflammatory Rasch-built Overall Disability Scale (I-RODS) (Van den Bergh et al., 2021). In the absence of a specific biomarker, current diagnostic methods are imperfect and false positive diagnoses occur with appreciable frequency (Kanbayashi et al., 2024). The range of differential diagnoses for CIDP is broad, spanning autoimmune, infectious, metabolic, and neoplastic neuropathies. In practice, however, the foremost challenge is often distinguishing CIDP from diabetic polyneuropathy, given the overlapping clinical and electrophysiological features (Cox and Gwathmey, 2021; Ercolini and Miller, 2009). The relationship between CIDP and diabetes mellitus (DM) is bidirectional and remains incompletely defined: some studies suggest a higher frequency of CIDP among people with diabetes, whereas others do not confirm this association (Bril et al., 2016; Laughlin et al., 2009; Rajabally et al., 2017; Rajabally et al., 2020). DM has also been proposed as a trigger of autoimmunity through the release of danger associated molecular patterns (DAMPs) that are ordinarily sequestered intracellularly (Block et al., 2022; Kroemer et al., 2024; Ma et al., 2024). What is clear is that DM can produce a demyelinating polyneuropathy even at early stages of metabolic disturbance, including prediabetes, through multiple mechanisms (Abraham et al., 2015; Dunnigan et al., 2013; Latov, 2011). A clinically relevant point is that demyelinating polyneuropathy in DM responds poorly to immunomodulatory therapy (Breiner et al., 2019), and concomitant DM in patients with CIDP is associated with lower odds of treatment success (Andrusiów et al., 2023).

Collectively, these observations underscore the need for new biomarkers and pathogenic insights in CIDP. This study aimed to analyse the intestinal bacterial and fungal communities (microbiota and mycobiota) and their interrelationships, as potential contributors to metabolic and immune dysregulation in CIDP.

## 2 Materials and methods

### 2.1 Characteristics of the study groups

This study enrolled 32 patients with classic or atypical CIDP (multifocal, motor-predominant or sensory-predominant) and 15 healthy controls without clinical and/or electrophysiological features of neuropathy. Participants were hospitalized at the Neurology Department of Wrocław Medical University between 2021 and 2024. Fourteen of the patients had diabetes mellitus (DM+), while eighteen patients did not (DM-). All patients in the CIDP cohort were newly diagnosed during their hospitalization and had not received any prior immunomodulatory or immunosuppressive therapy. The diagnosis of CIDP was established according to the EAN/PNS 2021 diagnostic criteria (Van den Bergh et al., 2021), which included performing the broad differential diagnostic workup recommended by those guidelines. At study entry, all patients underwent a detailed neurological assessment, including evaluation with standardized clinical scales. In addition, an extensive panel of ancillary investigations was performed, including lumbar puncture with CSF analysis, neuroimaging and laboratory tests. These procedures were part of a broad differential diagnostic work-up carried out in accordance with the above-mentioned guidelines. Absolute exclusion criteria were any immunomodulatory or immunosuppressive treatment and a change of diagnosis during the 1-year follow-up. The study protocol was approved by the Bioethics Committee at Wrocław Medical University (number 932/2021). Detailed characteristics of the population are presented in the Table 1.

**TABLE 1:** Characteristics of the CIDP group stratified by diabetes status (DM+ and DM−) and the control group.

Characteristics	CIDP DM+ group (*n* = 14)	CIDP DM- group (*n* = 18)	Control group (*n* = 15)
Mean age (range) (years)	61 (47−73)	65 (40−84)	57 (21−79)
Sex (male/female)	13/1	14/4	7/8
Disease duration (years)	1−8	0.5−26	−
Comorbidities (n)	3	6	9
Comorbidities (type)	Ischaemic stroke/Hyperthyroidism/Depression	HBV infection/Heart failure/ Chronic kidney disease/Rheumatoid arthritis/Hypothyroidism/Interstitial lung disease	Headaches/Normotensive hydrocephalus
Atypical CIDP (n)	2	6	–
INCAT scale (0–4/ 5–10)	13/1	15/3	–
CSF protein level (median) (mg/dL)	65.6	59.7	44

DM, diabetes mellitus; CIDP, chronic inflammatory demyelinating polyneuropathy; CSF, cerebrospinal fluid; HBV, hepatitis B virus.

### 2.2 Clinical and neurodiagnostic assessments: electrophysiology, neuroimaging, and CSF collection

Patients were routinely assessed using the Inflammatory Neuropathy Cause and Treatment (INCAT) scale (Breiner et al., 2014) and divided into two groups: those with mild disease (INCAT 0–4) and those with severe disease (INCAT 5–10). Neuroimaging was performed on all participants using brain magnetic resonance imaging (MRI) to evaluate potential central nervous system involvement and to rule out other neurological conditions. After imaging, a lumbar puncture was performed at the L3/L4 intervertebral space under sterile conditions to collect CSF, following standard clinical procedures. The CSF samples were analyzed for basic laboratory parameters, including cell count, total protein, and glucose levels, and were further evaluated for immunological markers and additional biomarkers relevant to CIDP.

The standard electrodiagnostic evaluation encompassed multiple nerve conduction parameters. More specifically, the following parameters were measured: distal latency (DL), compound motor action potential (CMAP) amplitude, motor nerve conduction velocity (MCV), F-wave latency (F-Lat), sensory nerve action potential (SNAP) amplitude, and sensory nerve conduction velocity (SCV). NCS were conducted in the median and ulnar nerves (upper extremities) and the tibial, peroneal and sural nerves (lower extremities). Sensory conduction was assessed using antidromic stimulation. Needle electromyography of the tibialis anterior muscle was performed to evaluate muscle innervation. All tests were carried out using a Viking Quest EMG system (version 10.0). To ensure consistency across subjects, we maintained standardized testing conditions. Identical electrode placements and fixed distances between stimulation and recording sites were used for each nerve. Stimuli were delivered at defined anatomical locations to obtain distal latency, amplitude, and conduction velocity. The stimulus pulse duration was set to 0.2 ms for motor fibers and 0.1 ms for sensory fibers. Ambient room temperature was controlled between 21 and 23 °C, while patient hand temperature was kept above 32 °C and foot temperature above 30 °C during examinations. All studies were administered by experienced, certified clinicians, adhering to the protocol described by Oh (2003) and in compliance with the 2021 EAN/PNS guidelines for CIDP (Van den Bergh et al., 2021).

### 2.3 DNA analysis

Genomic DNA was extracted from peripheral blood collected in EDTA tubes using the NucleoSpin Blood DNA Purification Kit (Macherey-Nagel, Germany) according to the manufacturer’s instructions. DNA concentration and purity were assessed using a DeNovix spectrophotometer (DeNovix Inc., United States), based on A260/280 and A260/230 ratios. Extracted DNA was used for *IL18* genotyping.

### 2.4 Genotyping of *IL18* promoter polymorphisms

Genotyping of *IL18* promoter polymorphisms (rs187238, rs1946518, rs1946519) was carried out with LightSNiP assays (TIB Molbiol, Germany). Polymerase chain reaction (PCR) amplification was performed on a LightCycler 480 II Real-Time PCR system (Roche Diagnostics, International AG, Rotkreuz, Switzerland). The thermal cycling program consisted of an initial denaturation at 95 °C for 10 min, followed by 45 cycles of 95 °C for 10 s, 60 °C for 10 s, and 72 °C for 15 s. This was followed by a high-temperature step at 95 °C for 30 s and a cool-down to 40 °C for 2 min, after which a gradual melting curve from 40 to 75 °C was generated. Genotypes were assigned by examining the characteristic melting temperature peaks and profiles according to the manufacturer’s instructions.

The distribution of *IL18* genotypes for the analyzed polymorphisms is presented in Supplementary Table 1.

### 2.5 SCFAs measurement in stool, serum, and CSF samples

SCFAs in stool samples were extracted and derivatized prior to analysis. In brief, 500 μL of a homogenized fecal sample was combined with 3 mL of 70% ethanol and thoroughly mixed, then centrifuged to remove particulate matter. Then, 500 μL of the clear supernatant was transferred to a fresh tube and the following derivatization reagents were added: 50 μL of internal standard (2-ethylbutyric acid, 200 mM in 50% aqueous methanol), 300 μL of pyridine (3% v/v in ethanol; Merck), 300 μL of 250 mM *N*-(3-dimethylaminopropyl)-*N*′-ethylcarbodiimide hydrochloride (Sigma-Aldrich) in ethanol, and 300 μL of 20 mM 2-nitrophenylhydrazine hydrochloride (Sigma-Aldrich) in ethanol. The mixture was then incubated at 60 °C for 20 min. To quench the reaction, 200 μL of 15% (w/v) sodium hydroxide in water (mixed with methanol at an 80:20 ratio, v/v) was added, and the sample was cooled to room temperature. The solution was subsequently extracted twice with 2 mL of 0.5 M phosphoric acid and 4 mL of diethyl ether, combining the ether layers from each extraction. The pooled organic phase, containing the SCFA hydrazide derivatives, was washed with water and then evaporated to dryness. Finally, the residue was reconstituted in 150 μL of methanol, yielding the prepared sample for chromatographic analysis.

Analysis of the derivatized SCFAs was performed by high-performance liquid chromatography (HPLC). A Waters 1525 Binary HPLC Pump coupled to a Waters 2489 UV/Visible Detector, with detection set at 400 nm was used. Chromatographic separation was achieved on a Shimadzu C18 reverse-phase column (5 μm particle size, 120 Å pore size, 250 × 4.6 mm) using a mobile phase of acetonitrile, methanol and water in a 30:16:54 ratio. The column temperature was maintained at 50 °C and the flow rate was 1.0 mL/min.

For serum and CSF specimens, the same extraction protocol was followed with slight modifications. Only 1 mL of 70% ethanol was used for these fluid samples, and the CSF was not centrifuged because it contains negligible particulate matter.

### 2.6 Amplicon sequencing

DNA was extracted from frozen fecal samples using the Maxwell RSC Faecal Microbiome DNA Kit, and DNA from frozen CSF samples was extracted using the Maxwell RSC Pathogen Total Nucleic Acid Kit. Isolation was carried out using the Maxwell RSC system. DNA concentration was determined by fluorometry (Quantus, Promega). Quality control of the prepared libraries was performed using the High Sensitivity D1000 ScreenTape System on the Agilent 4200 TapeStation system (Agilent Technologies). For library preparation, 1 ng of input DNA was used per sample. Subsequently, library concentrations were reassessed fluorometrically (Quantus, Promega), diluted in accordance with the QIAseq 16S/ITS Panel Handbook (Qiagen), pooled, and sequenced on the MiSeq platform (Illumina) using the MiSeq Reagent Kits v3 (600 cycles) for paired-end sequencing. Amplicon sequencing of the V3V4 region of the 16S rRNA gene and the ITS1 region was performed using the QIAseq 16S/ITS Panel Region Kit (Qiagen) and the MiSeq platform (Illumina).

### 2.7 Metagenomic and statistical analysis

Metagenomic analysis was performed using QIIME 2 software (version 2024.10) (Bolyen et al., 2019). The data were imported using *q2-tools*, after which the sequences of the V3V4 region were extracted, and primer sequences were removed from the reads using *q2-cutadapt* (Martin, 2011). Using *q2-demux*, the quality of the acquired sequences was evaluated, and the length of overlap was estimated. Next, the sequences were trimmed, random error effects were eliminated, and the reads were dereplicated using *q2-dada2* (Callahan et al., 2016). Using *q2-feature-classifier*, the V3V4 region of the 16S rRNA gene was extracted from the GreenGenes2 database reads (version 2024.09) (Bokulich et al., 2018; McDonald et al., 2024), and then a naïve Bayes classifier was created and used to assign appropriate taxonomic labels to the amplicon sequence variants (Bokulich et al., 2018; Pedregosa et al., 2018). Fungal (ITS1) reads were extracted with q2-itsxpress (Einarsson and Rivers, 2024), then processed analogously, with denoising performed in QIIME 2 and taxonomic classification against a fungal reference database (UNITE, QIIME release, fungi, release date 2025-02-19, version 10.0), using a classifier trained on the corresponding ITS1 region.

Additionally, for CSF samples (due to low sample biomass), the decontamination process was performed using the *IsNotContaminant* function from the decontam package available in R (software version 4.4.1, decontam package version 1.24.0) (Davis et al., 2017).

Before diversity analysis, the amplicon sequence variant (ASV) tables underwent rarefaction using q2-feature-table (Einarsson and Rivers, 2024), which resulted in the exclusion of 1 stool microbiome sample, 2 CSF microbiome samples, and 17 stool mycobiome samples.

The alpha-diversity metrics [observed ASVs, Shannon’s diversity index, Simpson’s diversity index, Faith’s phylogenetic diversity (Faith’s PD)] (Faith, 1992; Shannon, 1948; Simpson, 1949) and beta-diversity metrics (Bray-Curtis statistic, Jaccard similarity index, Euclidean distance, unweighted and weighted UniFrac) (Hamady et al., 2010; Jaccard, 1908; Legendre and De Cáceres, 2013; Lozupone and Knight, 2005; Lozupone et al., 2007; McDonald et al., 2018; Sorensen, 1948) were calculated using *q2-diversity*. To compare diversity values between groups and to investigate statistically significant differences and correlations between microbiota and mycobiota and clinical/laboratory data, statistical tests were performed. For alpha-diversity metrics, the Kruskal-Wallis test (KW) (Kruskal and Wallis, 1952) was applied for categorical metadata, and Spearman’s rank correlation test (Pearson, 1895; Spearman, 1987) was used for numerical metadata. For beta-diversity metrics, permutational multivariate analysis of variance (PERMANOVA) was used for categorical metadata (Anderson, 2001) and for numerical metadata, the Mantel test (MacLeod, 1967) was applied. The Benjamini-Hochberg procedure was used to control the false discovery rate (FDR) for pairwise tests (adjusted *p*-values are defined as q-values in the manuscript). For the beta-diversity metrics, principal coordinates analysis (PCoA) was performed using *q2-diversity* (Halko et al., 2010).

NGS sequencing and downstream processing yielded alpha- and beta-diversity indices, as well as taxonomic profiles at multiple taxonomic levels (order, family, genus, species). These data were compared with clinical assessment results (presence of diabetes, CIDP phenotype, disability severity according to the INCAT scale), nerve conduction studies (tibial, peroneal, sural, median, ulnar), SCFA measurements in stool/serum/CSF (acetic, propionic, butyric, valeric, isovaleric acids), CSF protein levels, and genotyping of *IL18* polymorphisms (rs187238, rs1946518, rs1946519). Samples without metadata were excluded from the statistical tests. Genotyping results were not available for two patients due to technical limitations.

A taxonomic composition analysis was conducted using ANCOM-BC2 (Analysis of Composition of Microbiomes with Bias Correction 2) available in R (software version 4.4.1, ANCOMBC package version 2.6.0) (Lin and Peddada, 2020). *P*-values were Holm-adjusted for multiple testing (q-values in the manuscript). Potential contaminants were addressed at several levels. We closely examined ASVs classified as typical reagent or environment-associated taxa that occurred at very low abundance. If an ASV’s distribution was consistent with contamination (e.g., found in low-density CSF samples but absent in stool), it was removed from further analysis.

## 3 Results

The final datasets of stool samples were dominated by Bacteria (with a small contribution of Archaea) and a much sparser fungal signal. In CSF, sequencing indicated the presence of Bacteria, whereas Archaea were not detected. Moreover, analysis of the CSF mycobiome did not yield any non-contaminant ASVs. Consequently, no further diversity or taxonomic analyses were performed for CSF fungi. The data suggest that, within the sensitivity of this protocol, CSF is effectively sterile for fungi in both CIDP patients and controls.

In the subsections below, we present statistically significant differences and correlations between alpha-/beta-diversity metrics and clinical, electrophysiological and laboratory data. In the text, we report strong (*R* ≥ 0.7) or moderate (*R* ≥ 0.5) correlations, with the diversity metrics for which statistical significance was found indicated in parentheses.

### 3.1 Fecal microbiota

**FIGURE 1:**
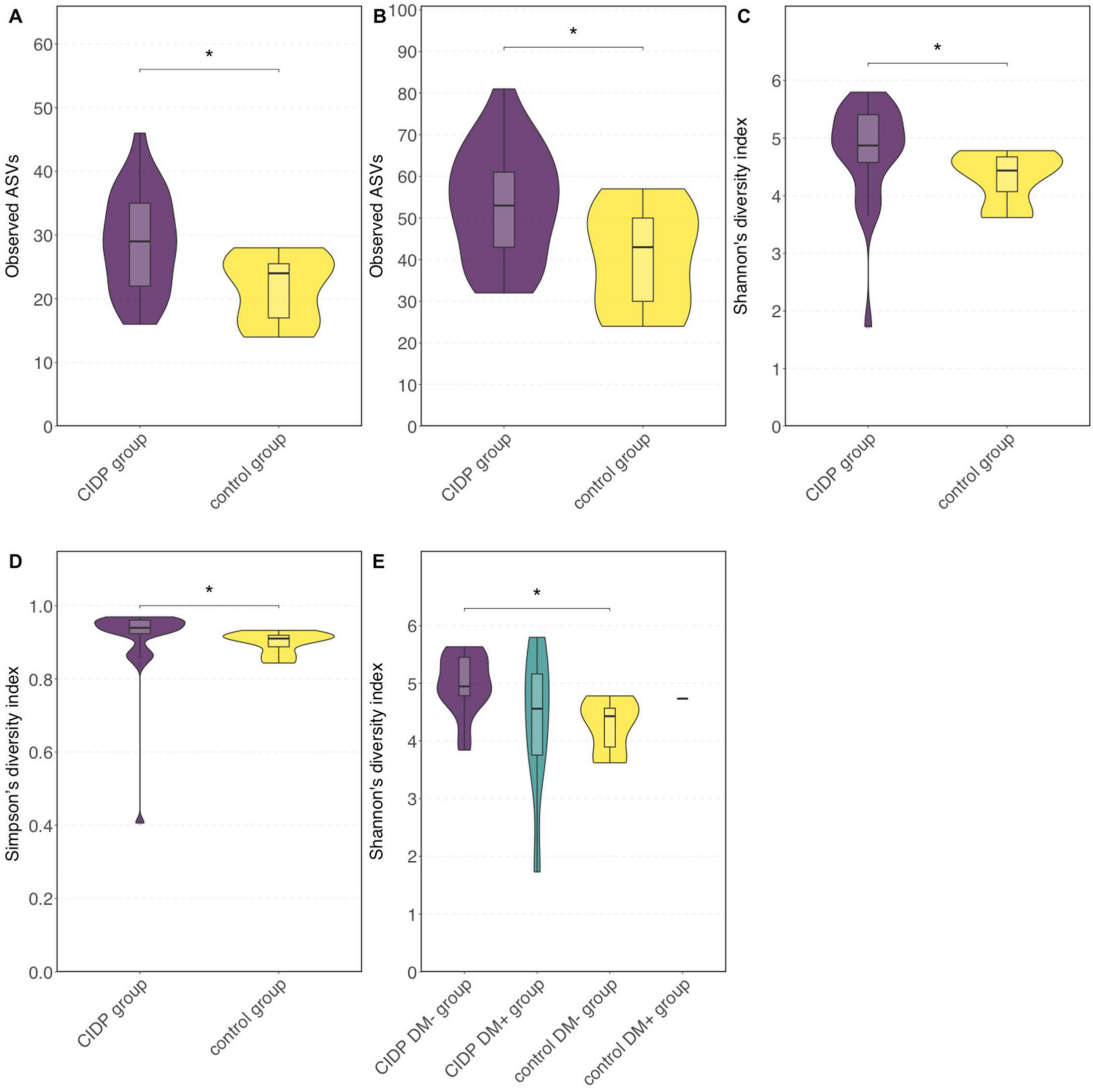
Number of observed ASVs in the CIDP and control groups at the taxonomic levels of order **(A)** and family **(B)**. Shannon’s diversity index **(C)** and Simpson’s diversity index **(D)** diversity indices in the CIDP and control groups at the genus level. **(E)** Shannon’s diversity index for subgroups of CIDP and control groups, with type 2 diabetes (DM+) and without diabetes (DM-) at the genus level. “*” indicate *p* < 0.05.

**FIGURE 2:**
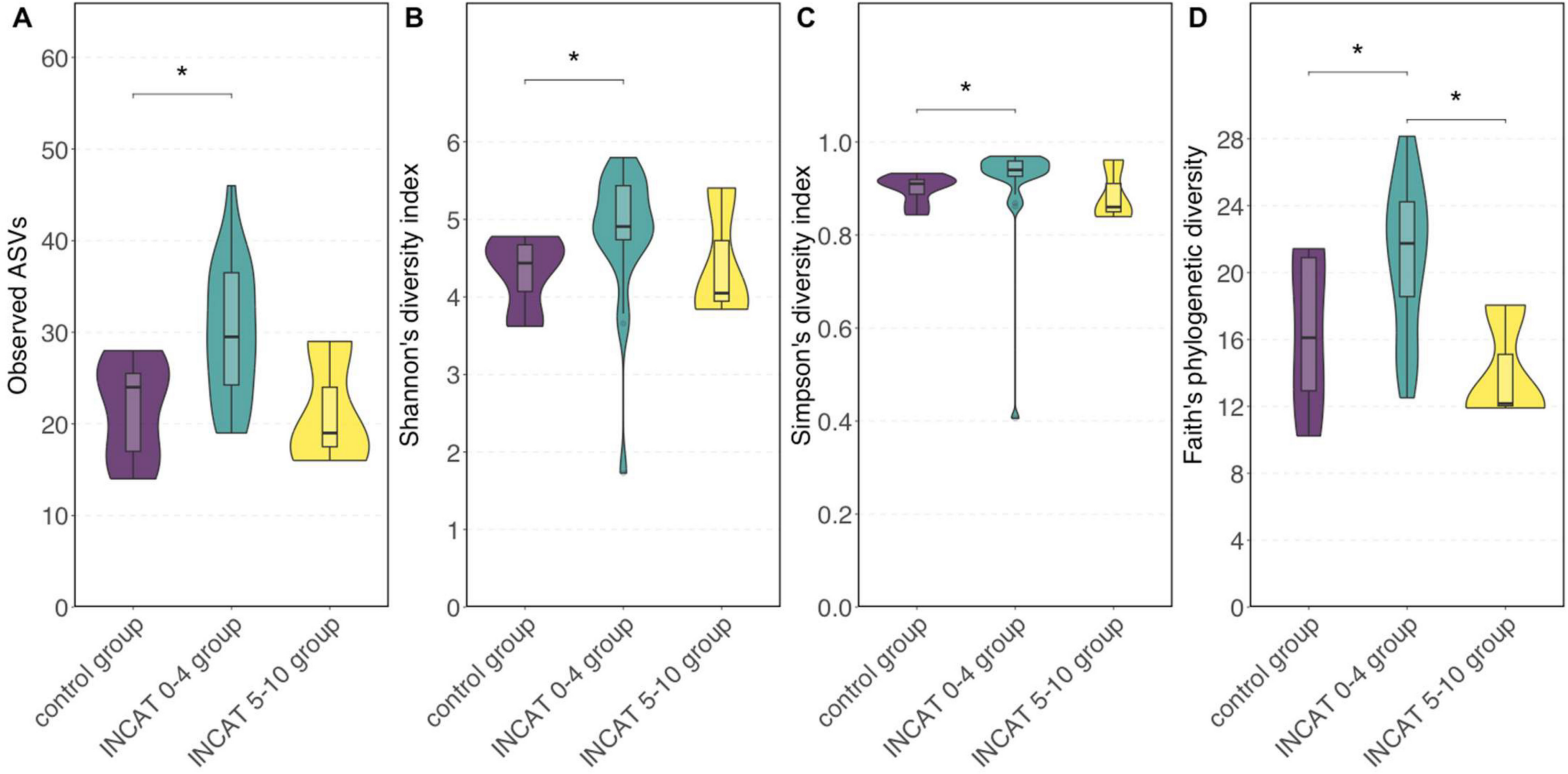
Number of observed ASVs in the INCAT groups at the order level **(A)**; Shannon’s diversity index **(B)** and Simpson’s. Diversity index **(C)** diversity indices at the genus level; Faith’s PD at the species level **(D)**. “*” indicate *p* < 0.05.

**FIGURE 3:**
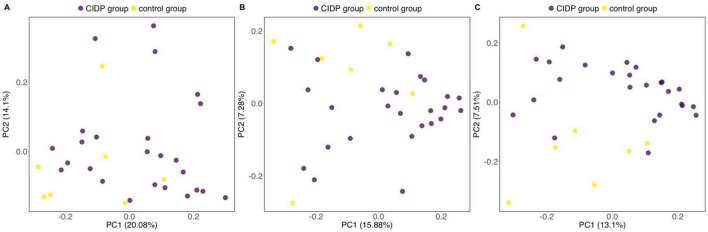
PCoA based on the Jaccard similarity index in the CIDP and control groups at the taxonomic levels of order **(A)**, genus **(B)** and species **(C)**.

When comparing the CIDP and control groups within individual samples, we found that in the CIDP group there were significantly more observed ASVs aggregated at the order level and family level (*p*, *q* = 0.0224, *p*, *q* = 0.0379, respectively). Moreover, there were also significantly higher numbers and frequencies of ASVs, and higher sample homogeneity at the genus-level diversity (Shannon’s diversity index, *p*, *q* = 0.0240; Simpson’s diversity index, *p*, *q* = 0.0381) (Figures 1A–D). A significant difference in the number and frequencies of ASVs at the genus-level diversity (Shannon’s diversity index, *p* = 0.004097, *q* = 0.024584) was still present when only patients without concomitant diabetes were considered (Figure 1E). In subgroup analyses according to disability severity on the INCAT scale (0–4 vs. 5–10), we detected in mildly affected patients compared with healthy controls significantly higher number of observed ASVs aggregated at order level (*p* = 0.0123, *q* = 0.0371), significantly higher numbers and frequencies of ASVs, and higher sample homogeneity at genus level (Shannon’s diversity index, *p* = 0.0125, *q* = 0.0375; Simpson’s diversity index, *p* = 0.0144, *q* = 0.0433), and higher phylogenetic diversity at the species level (Faith’s PD, *p* = 0.0323, *q* = 0.0485). We also observed a similar difference between mildly affected and severely affected patients at the species level (Faith’s PD, *p* = 0.0192, *q* = 0.0485) (Figure 2). The above alterations in gut microbiota were also supported by beta-diversity metrics. Comparing the CIDP and control groups, we observed significant differences in community membership at the order level (Jaccard similarity index, *p*, *q* = 0.036), and in community composition and membership at the genus level (Bray-Curtis statistic, *p*, *q* = 0.017; Jaccard similarity index, *p*, *q* = 0.015) and species level (Bray-Curtis statistic, *p*, *q* = 0.023; Jaccard similarity index) between samples (Figure 3). Furthermore, statistically significant differences in the number of observed ASVs, or in fraction of unique ASVs after consideration of their number and phylogenetic relationship (Euclidean distance, *p*, *q* = 0.034; *p*, *q* = 0.005; weighted UniFrac *p*, *q* = 0.037).

**FIGURE 4:**
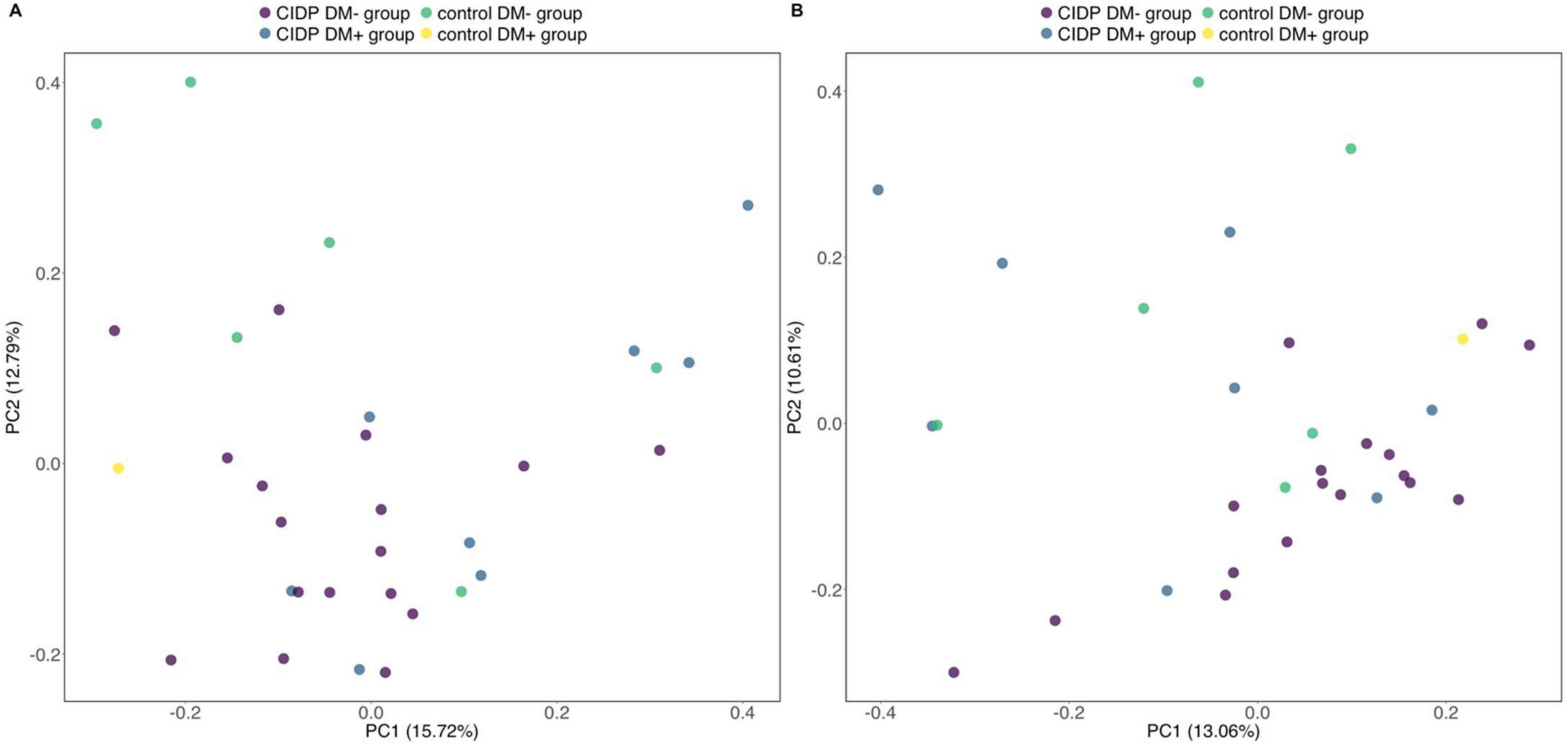
PCoA based on the Bray-Curtis statistic for subgroups with type 2 diabetes (DM+) and without diabetes (DM-), at the taxonomic levels of genus **(A)** and species **(B)**.

Significant differences in community membership were likewise seen between patients with classical (but not atypical) CIDP and controls at the level of order (Jaccard similarity index, *p* = 0.013, *q* = 0.0390) and species (Jaccard similarity index, *p* = 0.005, *q* = 0.0150); between mildly affected patients and controls at the level of genus (Jaccard similarity index, *p* = 0.003, *q* = 0.009) and species (Jaccard similarity index, *p* = 0.005, *q* = 0.015). In comparisons between CIDP DM+ and DM-, we observed significant differences in the number of observed ASVs, even after accounting for their frequencies between samples at the level of genus (Euclidean distance, *p* = 0.001, *q* = 0.0060; Bray-Curtis statistic, *p* = 0.002, *q* = 0.0120) and species (Euclidean distance, *p* = 0.006, *q* = 0.0360; Bray-Curtis statistic, *p* = 0.005, *q* = 0.0240 samples in observed unique ASVs, even with consideration of their abundance and phylogenetic relationships (Jaccard similarity index, *p* = 0.006, *q* = 0.0360; weighted UniFrac, *p* = 0.004, *q* = 0.0240) (Figure 4).

We found moderately strong, positive correlations between age and richness (observed ASVs) and genus-level diversity (observed ASVs, *p* = 0.0105; *R* = 0.5124; Shannon’s diversity index, *p* = 0.0031; *R* = 0.5785), as well as the species level (observed ASVs, *p* = 0.0056; *R* = 0.5482; Shannon’s diversity index, *p* = 0.0078; *R* = 0.5297). We also observed a positive correlation between age and genus-level diversity (Simpson’s diversity index, *p* = 0.0122; *R* = 0.5031).

For electrophysiological data, positive correlations were observed between tibial nerve F-Lat and the number and frequencies of observed ASVs within sample at the level of order (Shannon’s diversity index, *p* = 0.0133; *R* = 0.6034), family (Shannon’s diversity index, *p* = 0.0251; *R* = 0.5946), genus (Shannon’s diversity index, *p* = 0.0197; *R* = 0.5754) and species (Shannon’s diversity index, *p* = 0.0272; *R* = 0.5504); homogeneity of samples at order level (Simpson’s diversity index, *p* = 0.0476; *R* = 0.5018), and observed ASVs within sample at species level (observed ASVs, *p* = 0.0247; *R* = 0.5579). However, for peroneal nerve F-Lat we observed positive correlations only with the number and frequencies of observed ASVs within sample only at the order level (Shannon’s diversity index, *p* = 0.04242; *R* = 0.6833).

Negative, moderately strong correlations were found between ulnar nerve SNAP amplitude and number of observed ASVs, even after accounting their frequencies within samples, and homogeneity of samples at the genus level (observed ASVs, *p* = 0.0240; *R* = − 0.5024; Shannon’s diversity index, *p* = 0.0140; *R* = −0.5397; Simpson’s diversity index, *p* = 0.0042; *R* = −0.6112) and species (observed ASVs. *p* = 0.0122; *R* = −0.5489; Shannon’s diversity index, *p* = 0.0082; *R* = −0.5736; Simpson’s diversity index, *p* = 0.0058; *R* = −0.5939); between tibial nerve CMAP amplitude and diversity at the genus level (Simpson’s diversity index, *p* = 0.0150; *R* = −0.5113) and species level (Simpson’s diversity index, *p* = 0.0093; *R* = −0.5413), and with number and frequencies of observed ASVs within samples at species level (Shannon’s diversity index, *p* = 0.0132; *R* = −0.5198); between ulnar nerve SCV and homogeneity of samples at the species level (Simpson’s diversity index, *p* = 0.0245; *R* = −0.5010).

For laboratory data, we observed moderately strong, negative correlations between fecal propionic acid concentration and number of observed ASVs within samples or homogeneity of samples at the family level (observed ASVs, *p* = 0.0038; *R* = −0.5580; Simpson’s diversity index, *p* = 0.0109; *R* = −0.5000), and richness within samples at the genus level (observed ASVs, *p* = 0.0059; *R* = −0.5345) and species level (observed ASVs, *p* = 0.0208; *R* = −0.5005), even after accounting their phylogenetic relationship (Faith’s PD, *p* = 0.0017; *R* = −0.5938). For fecal acetic acid concentration, we also noted moderately strong correlation with number of observed ASVs within samples at the genus level (*p* = 0.0080; *R* = −0.5180) and species level (*p* = 0.0101; *R* = −0.5043); even with phylogenetic relationships of ASVs (Faith’s PD, *p* = 0.0029; *R* = 0.5700); and for fecal valeric acid concentration correlation with observed ASVs at the family level (*p* = 0.0052; *R* = −0.5516).

Other statistically significant differences/correlations, together with the corresponding *p*/*q*/*R*-values, are presented in Supplementary Table 2 for alpha diversity and in Supplementary Table 3 for beta diversity.

**FIGURE 5:**
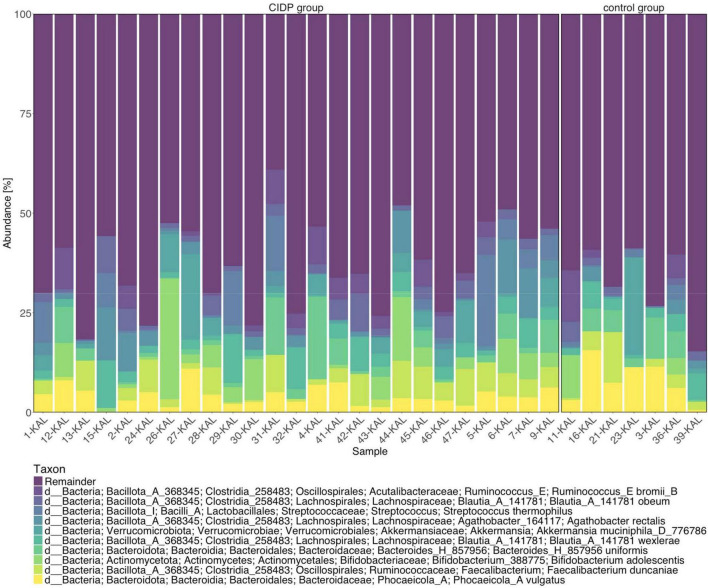
Barplot of the 10 most prevalent bacterial species of fecal microbiota.

In the taxonomic analysis, we identified Bacteria belonging to 73 orders, 151 families, 435 genera and 531 species (Figure 5). Archaea were represented by two methanogenic species (*Methanobrevibacter A smithii A* and *Methanosphaera stadtmanae*), with no significant differences in their prevalence or relative abundance between groups.

**FIGURE 6:**
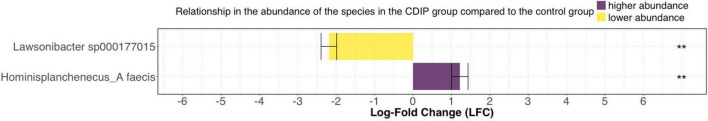
Log-fold change (LFC) for bacterial species showing statistically significant differences between the CIDP group and the control group. “**” indicate *q* < 0.01.

We observed statistically significant differences in the composition of the gut microbiota between the CIDP and control groups, as well as in subgroup analyses. The microbiota of CIDP patients was significantly enriched in bacteria of the order TANB77 (LFC = 3.9298, *p* = 1.99 × 10^–9^, *q* = 8.95 × 10^–8^) and the family CAG-508 (LFC = 3.9298, *p* = 1.97 × 10^–9^, *q* = 1.57 × 10^–7^); the same pattern was seen when only mildly affected patients (INCAT 0–4) (LFC = 3.8428, *p* = 4.0041 × 10^–6,^ *q* = 0.0001 and LFC = 3.8428, *p* = 4.7504 × 10^–6^, *q* = 0.0003, accordingly) or patients with classical CIDP were compared with controls (LFC = 3.9615, *p* = 2.6902 × 10^–6^, *q* = 0.0001, and LFC = 3.9615, *p* = 2.9853 × 10^–6^, *q* = 0.0002, accordingly). At the genus level, CIDP patients showed enrichment in *Hominisplanchenecus_A* (LFC = 1.2218, *p* = 2.73 × 10^–5^, *q* = 0.0060) and depletion of *Eubacterium_F* (LFC = −1.525301, *p* = 1.57 × 10^–6^, *q* = 0.0004) and *Thomasclavelia* (LFC = −1.799607, *p* = 0.0002, *q* = 0.0458). At the species level, two statistically significant differences were detected: *Hominisplanchenecus_A faecis* was more frequent in the CIDP group (LFC = 1.2218, *p* = 2.54 × 10^–5^, *q* = 0.0071), whereas *Lawsonibacter sp000177015* was less frequent (LFC = −2.1893, *p* = 4.81 × 10^–6^, *q* = 0.0014) (Figure 6).

**FIGURE 7:**
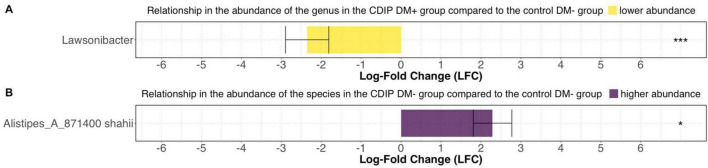
Log-fold change (LFC) for bacterial genus **(A)** and species **(B)** showing statistically significant differences between CIDP DM+ **(A)** and CIDP DM-(B) and controls DM- A. “*” indicate *p* < 0.05; “***” indicate *q* < 0.001.

When comparing CIDP DM- patients to DM− controls, we found a significantly higher prevalence of the family CAG-272 in the CIDP group (LFC = 2.9501, *p* = 8.62 × 10^–5^, *q* = 0.0056), and enrichment of the species *Alistipes_A_871400 shahii* (LFC = 2.289368, *p* = 0.0001, *q* = 0.0302). Moreover, CIDP DM+ patients in comparison to DM- controls showed a significant depletion of the genus *Lawsonibacter* (LFC = −2.352325, *p* = 0.0002, *q* = 0.0425) (Figure 7).

**FIGURE 8:**
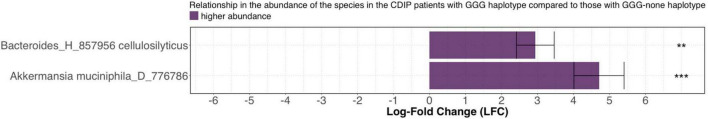
Log-fold change (LFC) for bacterial species showing statistically significant differences between CIDP patients carrying the GGG haplotype and those with non-GGG haplotypes. “**” indicate *q* < 0.01; “***” indicate *q* < 0.001.

**FIGURE 9:**
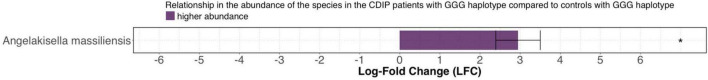
Log-fold change (LFC) for bacterial species showing statistically significant differences between CIDP patients carrying the GGG haplotype and controls carrying GGG haplotype. “*” indicate *q* < 0.05.

In the haplotype-based analysis of *IL18* polymorphisms, patients carrying the *GGG* haplotype (rs187238 *G*/rs1946518 *G*/rs1946519 *G*), compared with non-*GGG* carriers, had significantly greater abundances of the order Verrucomicrobiales (LFC = 4.7081, *p* = 6.76 × 10^–7^, *q* = 2.50 × 10^–5^), the family Akkermansiaceae (LFC = 4.7081, *p* = 9.84 × 10^–7^, *q* = 6.30 × 10^–5^), the genus *Akkermansia* (LFC = 4.7081, *p* = 9.87 × 10^–7^, *q* = 0.0002), and two species: *Bacteroides_H_857956 cellulosilyticus* (LFC = 2.9403, *p* = 3.84 × 10^–5^, *q* = 0.0089) and *Akkermansia muciniphila_D_776786* (LFC = 4.7081, *p* = 1.41 × 10^–6^, *q* = 0.0003) (Figure 8). Furthermore, CIDP patients with the *GGG* haplotype differed from *GGG* controls by an enrichment of the species *Angelakisella massiliensis* (LFC = 2.9468, *p* = 0.0002, *q* = 0.0425) (Figure 9).

All bacterial taxa that reached statistical significance, together with their *p*-values, are presented in Supplementary Table 4.

### 3.2 CSF microbiota

**FIGURE 10:**
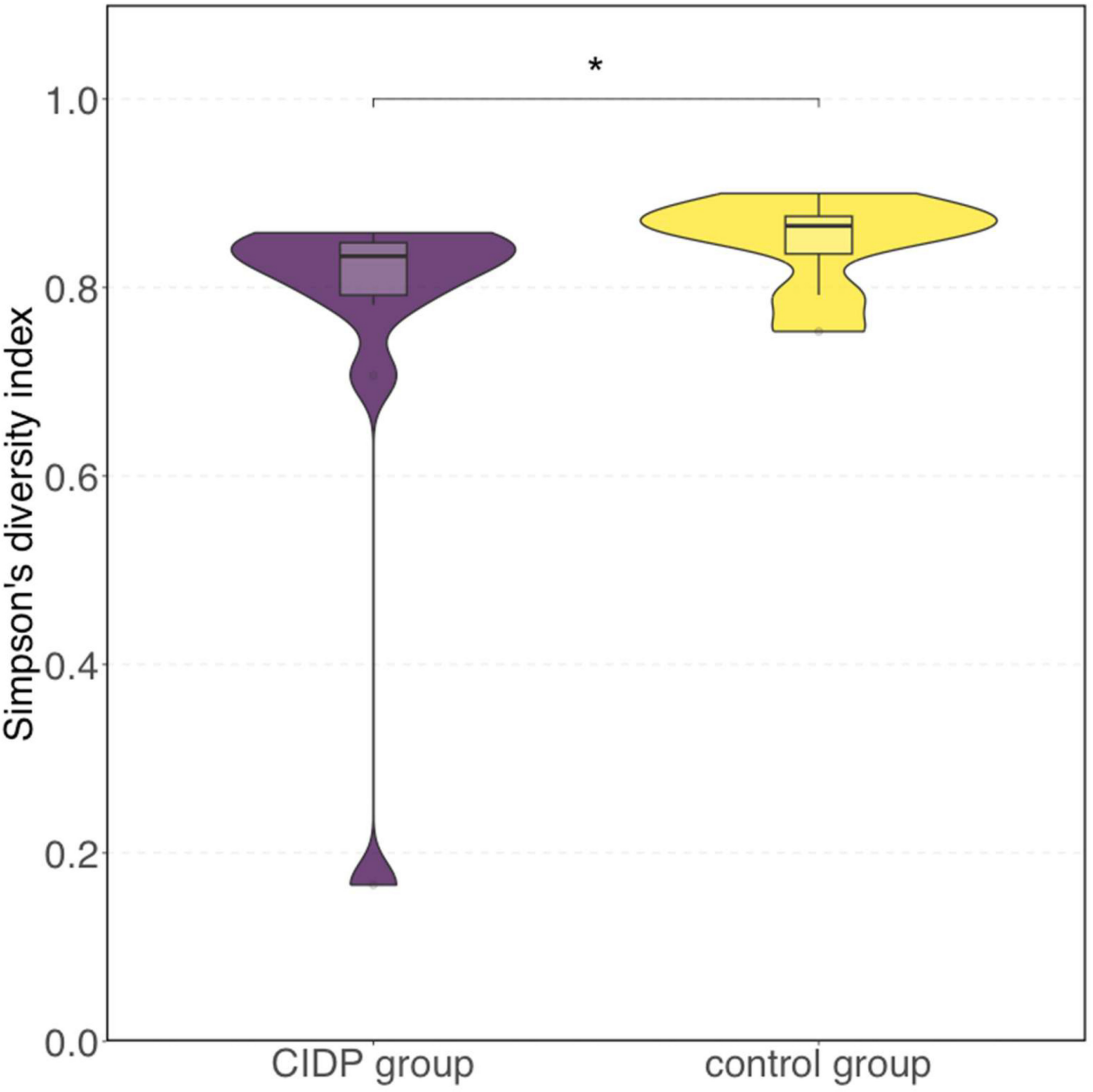
Simpson’s diversity index in the CIDP and control groups at the species level. “*” indicate *p* < 0.05

When analyzing differences between CIDP and control groups, we found significantly lower sample homogeneity in the CIDP group at the species level (Simpson’s diversity index, *p*, *q* = 0.0475) (Figure 10).

In analyses of electrophysiological parameters in relation to alpha diversity metrics, we found strong positive correlations between median nerve SNAP amplitude and number, and frequencies of ASVs within samples, and homogeneity of samples at all taxonomic levels (Shannon’s diversity index, order: *p* = 0.0065, *R* = 0.8571, family: *p* = 0.0065, *R* = 0.8571, genus: *p* = 0.0465, *R* = 0.7143, species: *p* = 0.0465, *R* = 0.7143; Simpson’s diversity index, order: *p* = 0.0009, *R* = 0.9286, family: *p* = 0.0020, *R* = 0.9048, genus: *p* = 0.0009, *R* = 0.9286, species: *p* = 0.0009, *R* = 0.9286); between median nerve SCV and homogeneity of samples at all taxonomic levels (Simpson’s diversity index, order: *p* = 0.0280; *R* = 0.7619, family: *p* = 0.0366; *R* = 0.7381, genus, species: *p* = 0.0465; *R* = 0.7143); between ulnar nerve SCV and samples homogeneity at the genus (Simpson’s diversity index, *p* = 0.0076; *R* = 0.7517) and species (Simpson’s diversity index, *p* = 0.0076; *R* = 0.7517) levels; between peroneal nerve CMAP amplitude and homogeneity of samples at the order (Simpson’s diversity index, *p* = 0.0059; *R* = 0.7671) and family (Simpson’s diversity index, *p* = 0.0035; *R* = 0.7945) levels, and numbers and frequencies of ASVs within samples at family level (Shannon’s diversity index, *p* = 0.0130; *R* = 0.7169); and between ulnar nerve SNAP amplitude and number of observed ASVs at the family level (*p* = 0.0130; *R* = 0.7170). In addition, a strong negative correlation was observed between ulnar nerve F-Lat and observed phylogenetic relationships of ASVs within samples at the species level (Faith’s PD, *p* = 0.0180; *R* = −0.7234).

With respect to beta diversity metrics, no statistically significant strong correlations with electrophysiological data were detected. Moderately strong positive correlations were found between median nerve CMAP amplitude and observed ASVs at the genus and species levels (Euclidean distance, *p* = 0.048; *R* = 0.5168 and *p* = 0.036; *R* = 0.5238, respectively); between ulnar nerve F-Lat and observed ASVs at the species level (Euclidean distance, *p* = 0.018; *R* = 0.5196); between for ulnar nerve CMAP amplitude and observed ASVs at all taxonomic levels (Euclidean distance, order: *p* = 0.002; *R* = 0.5995, family: *p* = 0.001; *R* = 0.6095, genus: *p* = 0.016; *R* = 0.5349, species: *p* = 0.014; *R* = 0.5423).

In the analysis of laboratory parameters in relation to alpha diversity metrics, we found strong positive correlations between CSF protein concentration and homogeneity of samples at the genus and species levels (Simpson’s diversity index, *p* = 0.0053; *R* = 0.7727 and *p* = 0.0053; *R* = 0.7727, respectively). Strong negative correlations were observed between serum acetic acid concentration and homogeneity of samples or number and frequencies of observed ASVs within samples at the order and family levels (Shannon’s diversity index, order: *p* = 0.0037; *R* = −0.7909, family: *p* = 0.0053; *R* = −0.7727; Simpson’s diversity index, order: *p* = 0.0098; *R* = −0.7364, family: *p* = 0.0085; R = −0.7455). No statistically significant strong correlations between laboratory variables and beta diversity metrics were identified. A moderately strong positive correlation was found between fecal acetic acid concentration and fraction of unique ASVs after consideration of their number and phylogenetic relationship (weighted UniFrac, *p* = 0.004; *R* = 0.6273).

Other statistically significant differences/correlations, together with the corresponding *p*/*q*/*R*-values, are presented in Supplementary Table 5 for alpha diversity and in Supplementary Table 6 for beta diversity.

**FIGURE 11:**
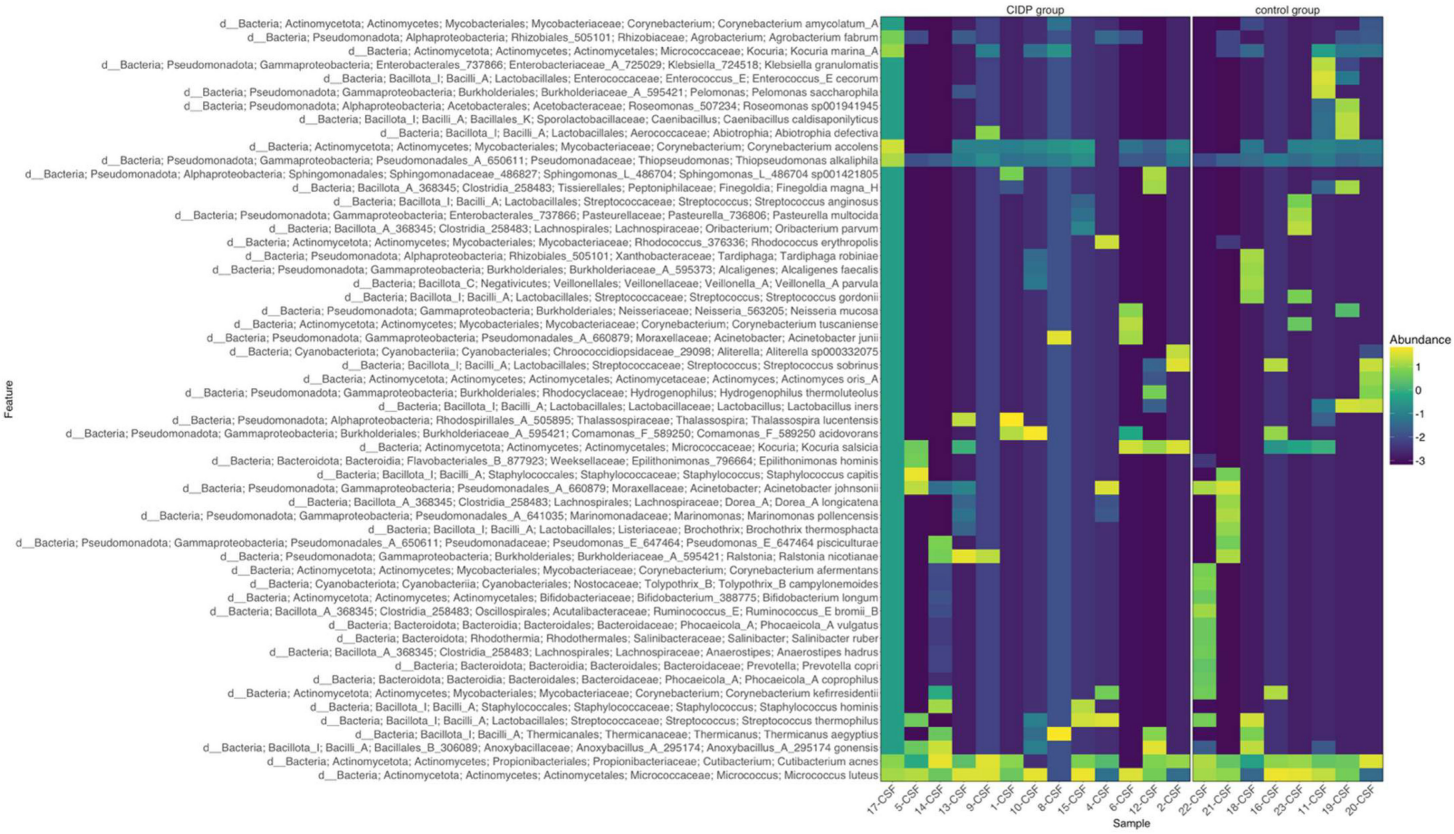
Heatmap of the occurrence of detected bacterial species in individual CSF samples.

In the taxonomic analysis, bacterial genetic material from 30 orders, 46 families, 62 genera and 56 species was detected (Figure 11). No archaeal taxa were identified.

There were no statistically significant differences in overall CSF microbiota composition between the total CIDP and control groups. However, significant differences emerged in subgroup analyses. Bacteria of the order Lactobacillales were less abundant in several subgroups: in mildly affected patients (INCAT 0–4) compared with controls (LFC = −4.2780, *p* = 0.0010, *q* = 0.0201); in the haplotype analysis between CIDP patients with the *GGG* haplotype and GGG controls (LFC = −4.4619, *p* = 0.0011, *q* = 0.0210), as well as between CIDP patients with *GGG* and non-*GGG* haplotypes (LFC = −4.4619, *p* = 0.0011, *q* = 0.0210); and between CIDP patients with the non-*CTT* haplotype and non-*CTT* controls (LFC = −5.7223, *p* = 0.0007, *q* = 0.0122). In addition, non-*CTT* patients had a reduced abundance of the family Streptococcaceae compared with non-CTT controls (LFC = −5.9338, *p* = 0.0001, *q* = 0.0011), and, relative to patients with the *CTT* haplotype, showed a further depletion of the genus *Streptococcus* (LFC = −5.116428, *p* = 0.0010, *q* = 0.0157).

### 3.3 Fecal mycobiota

Compared with the bacterial microbiota, statistical analyses of associations between fecal fungi and clinical/laboratory variables yielded far fewer statistically significant findings. No significant differences were detected between categorical clinical data and diversity metrics. However, statistically significant correlations were found between alpha and beta diversity metrics and clinical variables at all taxonomic levels.

For electrophysiological parameters, analyses of alpha diversity metrics showed strong positive correlations between peroneal nerve MCV and number of observed ASVs at the order level (*p* = 0.0053, *R* = 0.7224) and observed ASVs and their phylogenetic relationships at the species level (Faith’s PD, *p* = 0.0014; *R* = 0.7873) within samples. Strong negative correlations were observed between tibial nerve F-Lat and samples homogeneity or number and frequencies of observed ASVs within samples across all analyzed taxonomic levels (Shannon’s diversity index, order, family, genus, species: *p* = 0.0280, *R* = −0.7619; Simpson’s diversity index, order, family, genus, species: *p* = 0.0149, *R* = −0.8095). None of the analyzed parameters showed equally strong associations with beta diversity metrics. A positive, moderately strong correlation was found between tibial nerve F-Lat and fraction of unique ASVs with consideration of their phylogenetic relationship at the species level (unweighted UniFrac, *p* = 0.003; *R* = 0.50301) between samples.

A strong negative correlation between observed ASVs and their phylogenetic relationships and CSF propionic acid concentration at species level (Faith’s PD, *p* = 0.0024, *R* = −0.7407) was observed. In addition, statistically significant, moderately strong negative correlations were found between serum acetic acid concentration and number of observed ASVs within samples at all taxonomic levels (order: *p* = 0.0223, *R* = −0.5839, family, genus, species: *p* = 0.0187; *R* = −0.5974), and observed ASVs and their phylogenetic relationships at the species level (Faith’s PD, *p* = 0.0127; *R* = −0.6250); between CSF propionic acid concentration and number of observed ASVs at all taxonomic levels (order: *p* = 0.0315, *R* = −0.5749, family, genus, species: *p* = 0.0289, *R* = −0.5824); and between CSF acetic and butyric acid concentrations and observed ASVs and their phylogenetic relationships at the species level (Faith’s PD, *p* = 0.0470, *R* = −0.5385 and *p* = 0.0116, *R* = −0.6513, respectively). Moderately strong positive correlations were observed between CSF protein concentration and homogeneity of samples across all taxonomic levels (Simpson’s diversity index, order: *p* = 0.0428, *R* = 0.5286, family, genus, species: *p* = 0.0445; *R* = 0.5250).

Other statistically significant differences/correlations, together with the corresponding p/q/R values, are presented in Supplementary Table 7 for alpha diversity and in Supplementary Table 8 for beta diversity.

**FIGURE 12:**
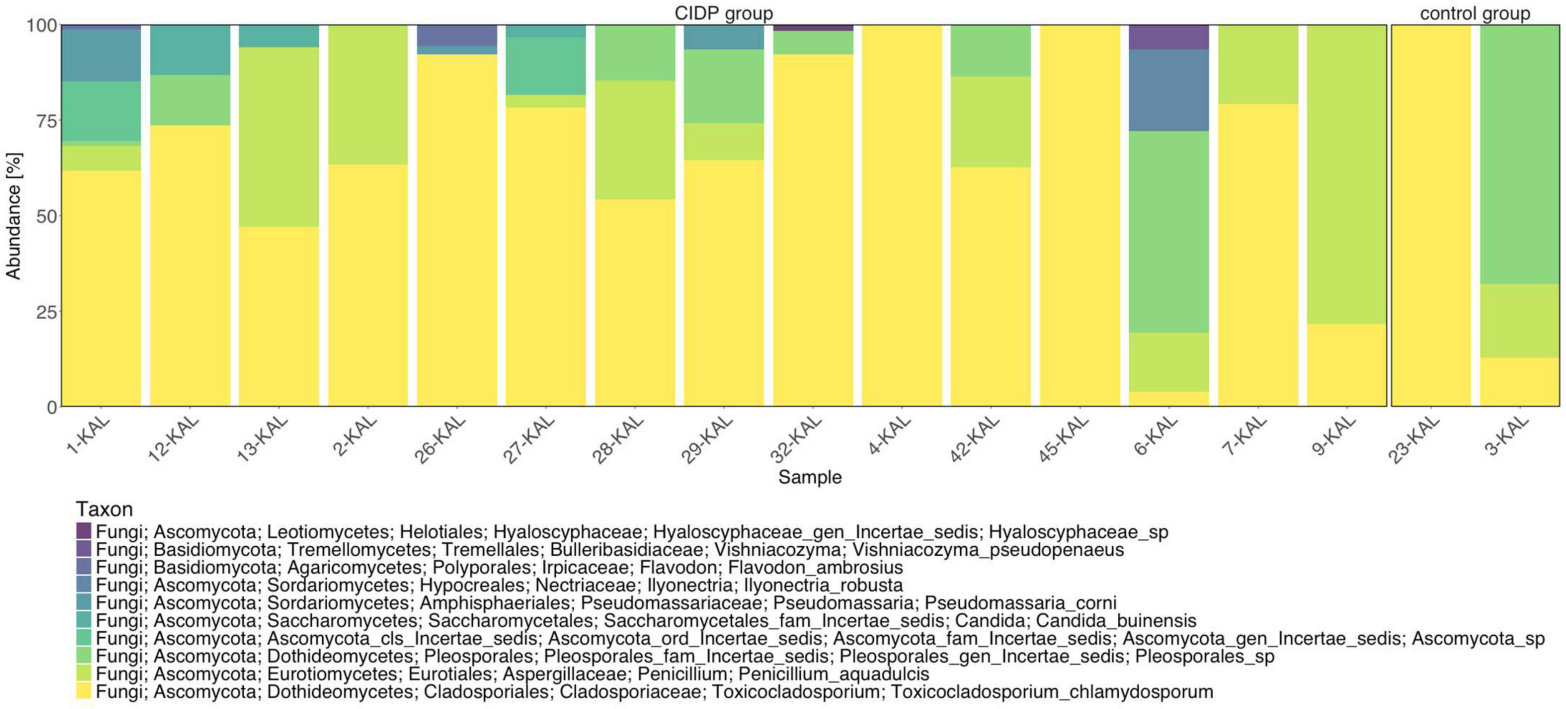
Bar plot of the percentage of detected fungal species in individual fecal samples.

In the taxonomic analysis, we identified 10 fungal orders, 10 families, 10 genera and 10 species (Figure 12). No statistically significant associations were detected at any taxonomic level.

## 4 Discussion

Despite the long-standing and steadily growing scientific interest in the microbiota, particularly in the context of autoimmune diseases, data on its relationship with CIDP remain very limited. One of the first observations in this field was made by Koszewicz et al. (2021b), who reported increased fecal calprotectin levels in patients with CIDP, which may reflect broadly understood inflammatory disturbances within the intestinal wall, although secondary dysautonomia cannot be excluded as a contributing factor. Of the two publications that have so far directly addressed the gut microbiota in CIDP, Svačina et al. (2023) demonstrated increased fecal microbiota alpha diversity in CIDP, with enrichment in Firmicutes (Ruminococcaceae and Lachnospiraceae) that produce SCFAs. Fu et al. (2023) in turn, described dysbiosis in CIDP characterized by an increased abundance of genes associated with opportunistic and potentially pathogenic bacteria, as well as a disturbed bile acid profile. Against this background, our study provides a more comprehensive view of how the intestinal microbial environment (both bacteria and fungi) is linked to the clinical aspects of CIDP. We confirmed previous reports of increased gut microbiota alpha diversity in CIDP and demonstrated that higher diversity indices correlate with worse electrophysiological parameters. Additionally, we demonstrated shifts in beta diversity, including in phylogenetic metrics (weighted UniFrac). Taken together, our results suggest multidimensional disturbances of the intestinal environment, affecting not only species abundance and diversity but also phylogenetic structure. In other words, greater alpha diversity was associated with both demyelinating and axonal conduction impairments. In contrast, correlations between beta diversity metrics and NCS parameters, although statistically significant, were much weaker. Within the CIDP group, we found increased abundance of *Hominisplanchenecus_A faecis* and reduced abundance of *Lawsonibacter sp000177015*. *Hominisplanchenecus_A faecis*, a member of the Lachnospiraceae family first described in 2022 by Afrizal et al. (2022), fits well with earlier CIDP microbiota findings, which highlighted SCFA-producing members of Lachnospiraceae. The previous studies did not resolve this signal to the species level. Emerging data already suggest a potential link between this genus and neurological disease. Zhang et al. (2025) identified genus *Hominisplanchenecus* as a candidate prognostic biomarker in aneurysmal subarachnoid hemorrhage. The second taxon, *Lawsonibacter sp000177015* from the Oscillospiraceae family, has not previously been associated with neurological or systemic autoimmune diseases. However, work by Guo et al. (2024), and Yang Y. et al. (2023) indirectly suggests a potentially beneficial effect of *Lawsonibacter sp000177015* on intestinal function.

We observed significant differences in diversity metrics across multiple subgroup comparisons. Diversity metrics differed between patients with (DM+) and without diabetes (DM−). We also observed differences when comparing mildly affected patients (INCAT 0–4) and either healthy controls or severely affected patients. Additionally, patients with the classical CIDP phenotype showed diversity metric shifts compared to healthy controls. Taxonomic analysis confirmed specific dysbiotic shifts and highlighted several significant differences. Compared with the control group, bacteria of the genus *Lawsonibacter* were significantly depleted in CIDP DM+, whereas in CIDP DM−, we found a significantly higher prevalence of *Alistipes_A_871400 shahii*, a species with an as yet unclear role in human physiology and pathophysiology (Parker et al., 2020). Moreover, the family *CAG-508* was significantly more frequent in patients with mild disease and in those with classical CIDP compared with controls. These findings represent some of the first observations that may cautiously suggest a potentially distinct pathogenesis in CIDP subgroups. Alternatively, they may provide preliminary evidence that the gut microbiota can modulate the clinical course of the disease.

In the interplay between the gut microbiota and the host, IL-18 probably represents one of the key mediators. In our cohort, microbiota composition differed significantly by *IL18* haplotype. CIDP patients with the *GGG* haplotype showed an enrichment of two species: *Bacteroides_H_857956 cellulosilyticus* and *Akkermansia muciniphila_D_776786*; compared to patients without the *GGG* haplotype. These two species are intensively studied and increasingly regarded as promising next-generation probiotic candidates, although their roles in neurological disease remain unknown (Gao et al., 2024; Jian et al., 2023). In the comparison between CIDP patients and controls, both carrying the *GGG* haplotype, the CIDP group was enriched in *Angelakisella massiliensis*. This species (first cultured in 2017), has so far not been convincingly linked to human pathology (Mailhe et al., 2017). Regardless of the exact functional relevance of these individual taxa, their association with *IL18* haplotypes in our data underscores the importance of IL-18 in shaping the gut microbiota. However, we did not observe more profound correlations between these IL18-related microbial patterns and the clinical phenotype of CIDP.

To complement the above observations, we also assessed SCFA concentrations and their relationships with laboratory and clinical parameters. Most studies suggest that SCFAs exert beneficial effects within the gut lumen (supporting intestinal barrier integrity) and have immunomodulatory, predominantly anti-inflammatory actions (Trend et al., 2021). Silva et al. (2020) conclude that SCFAs are predominantly beneficial, although many of the underlying data come from animal models and cannot be directly extrapolated to human health. Cantoni et al. (2022) demonstrated beneficial effects of SCFA supplementation and reduced SCFA levels in a prototypical demyelinating disease (MS). They also showed that autoimmune diseases are characterized by an extensive network of interactions between the microbiome, metabolome and immune system. In our cohort, we observed a negative correlation between fecal SCFA levels and gut bacterial diversity indices. In addition, we found, in CIDP group, reduced abundance of *Eubacterium_F*, a genus that includes known SCFA producers (Mukherjee et al., 2020). When considered together with our other findings (higher gut microbiota diversity in CIDP), these observations could suggest a possible role for reduced SCFAs availability or a decrease in SCFAs producing bacteria. Such changes might represent one of several factors contributing to CIDP pathogenesis. However, this interpretation remains highly speculative. Any causal relationship between SCFAs related alterations and CIDP will need to be tested and verified in future, appropriately powered studies.

Compared with the bacterial microbiota, the human fungal mycobiota remains much less well characterized. In a small study including six patients with CIDP, no differences in alpha diversity or significant species-level changes were detected (Verhasselt et al., 2023). By contrast, studies assessing the mycobiome in MS have demonstrated marked alterations, including increased fungal alpha diversity (Shah et al., 2021; Yadav et al., 2022). In our analysis, we did not detect significant differences in mycobiome diversity metrics between CIDP patients and controls, but we did observe several correlations with clinical variables. Overall, our data suggest a shift of the gut mycobiota toward a less diverse community with reduced evenness and a predominance of closely related, dominant taxa as peripheral nerve demyelination becomes more pronounced. Furthermore, we found a negative correlation between serum and CSF SCFAs levels and fungal alpha diversity across all taxonomic levels. This pattern may indicate displacement of SCFA-producing bacteria as fungal expansion progresses in the gastrointestinal tract.

The final aspect we investigated was the microbial genetic material in CSF. In our analysis, we detected dozens of bacterial taxa in CSF samples. However, we did not find statistically significant differences in CSF taxonomic composition between CIDP patients and controls. Bacteria of the order Lactobacillales were less frequently detected in several patient subgroups, including mildly affected individuals (INCAT 0–4) and carriers of the *GGG* haplotype. Additionally, genus *Streptococcus* was less abundant in patients with the non-*CTT* haplotype compared with *CTT* carriers. Diversity analysis revealed a significant reduction in species-level alpha diversity in the CIDP group. It also revealed that as CSF microbial diversity decreases, NCS abnormalities become more pronounced, an inverse relationship to that observed for fecal microbiota diversity. Among all the parameters analyzed, our finding of a significant deterioration in median nerve sensory conduction with decreased CSF alpha diversity stands out as particularly noteworthy. Previous studies have shown that the median nerve’s sensory fibers are especially susceptible to demyelination in CIDP. Median nerve SNAP amplitudes and CV are often more impaired (with a “sural-sparing” pattern of low median SNAP but normal sural SNAP) (Shibuya et al., 2022). Nevertheless, previous studies strongly support the notion that CSF is normally sterile and interpret low-level sequencing reads as contamination or as artifacts arising from insufficient filtering of sequencing data (Kang et al., 2020; Pandey et al., 2023; Ward et al., 2025). In our opinion, the sequences detected in CSF do not indicate true bacterial colonization. Instead, at least part of the detected genetic material may represent nucleic acid fragments translocated from the gut into the bloodstream and crossing a compromised blood–brain barrier. This interpretation is supported by the positive correlations we observed between CSF diversity metrics and NCS abnormalities. The greater the amount of genetic material of microorganisms that crosses the intestinal and blood–brain barrier, damaged by autoimmunity, the more pronounced the peripheral nerves damage. This hypothesis, however, requires further dedicated studies directly assessing blood–brain barrier integrity in CIDP.

## 5 Limitations and future directions

The authors are aware of several important limitations of the present study. The patient cohort was relatively small. Therefore, the observed findings cannot be directly translated into routine clinical practice. Nevertheless, this is one of the first studies to characterize the microbiota in a rare disease such as CIDP, confirming and extending previous preliminary reports. Moreover, microbiota composition and SCFA levels were assessed at a single time point, so all reported relationships between diversity metrics, metabolite concentrations and clinical or neurophysiological measures are correlational and do not allow causal inferences. In our view, this work should serve as a starting point for larger, multicentre studies. Such studies should explore the relationships between gut microorganisms, their metabolites, immune responses and the integrity of the blood–brain and blood–CSF barriers. The observed alterations suggest a promising avenue for developing targeted therapeutic interventions. Our findings provide a rationale for future clinical trials investigating whether the administration of specific probiotic strains could restore intestinal homeostasis and serve as an effective adjunctive therapy to standard immunosuppressive treatments. In addition, a logical next step will be to investigate the pharmacological potential of administering bacterial metabolites that are likely to have beneficial effects, such as SCFAs. Exploring the modulation of the neuroinflammation through such microbial based strategies may ultimately lead to more personalized and comprehensive management of CIDP.

## 6 Conclusion

CIDP appears to be another disease entity in which the intestinal microbial environment is likely to play an important role. In this study, we demonstrated significant shifts in the diversity and abundance of the gut microbiota and mycobiota with respect to clinically relevant reference measures (including NCS and INCAT). We confirmed previous reports of increased gut microbiota alpha diversity in CIDP. We also found that diversity metrics and the prevalence of certain taxa may differ significantly between CIDP subgroups defined by the presence of diabetes or by the clinical course. Furthermore, we identified correlations between parameters of peripheral nerve damage indicating that more intense demyelination process is associated with increased diversity of the gut microbiota and a decrease in gut mycobiota. The statistically significant and strong correlations (in some cases *R* > 0.9) between CSF diversity metrics and clinical data may suggest clinically relevant disruption of the intestinal and blood–brain barriers. Such disruption could allow DNA molecules to enter the CSF with further consequences within the central nervous system. Given the small sample size, all results must be interpreted with considerable caution. The observed associations should be verified in future studies to assess potential causal relationships.

## Data Availability

The datasets presented in this article are not readily available due to privacy and ethical restrictions. Requests to access the datasets should be directed to the corresponding author, szymon0946@wp.pl.
